# Non-Invasive Recording of Ocular-Following Responses in Children: A Promising Tool for Stereo Deficiency Evaluation

**DOI:** 10.3390/jcm13061596

**Published:** 2024-03-11

**Authors:** Aleksandar Miladinović, Christian Quaia, Miloš Ajčević, Laura Diplotti, Paola Michieletto, Agostino Accardo, Stefano Pensiero

**Affiliations:** 1 Department of Ophthalmology, Institute for Maternal and Child Health—IRCCS “Burlo Garofolo”, 34137 Trieste, Italy; 2 Laboratory of Sensorimotor Research, National Eye Institute, National Institutes of Health, Department of Health and Human Services, Bethesda, MD 20892, USA; 3 Department of Engineering and Architecture, University of Trieste, 34127 Trieste, Italy

**Keywords:** ocular-following response, stereopsis, video oculography, eye tracking

## Abstract

**Background:** The ability to merge the two retinal images to perceive depth (stereopsis) plays an important role in human vision. Its proper development requires binocular alignment and good visual acuity in both eyes during childhood. Because treatments are more effective when applied early, early diagnosis is important. Unfortunately, assessing stereo deficiencies in infants and young children remains challenging. Recently, it has been shown that ocular-following responses (OFRs; reflexive, short-latency eye movements induced by the sudden motion of a large textured pattern) are sensitive to changes in interocular correlation, making them potentially useful for stereo deficiency assessments. To test this hypothesis, we measured OFRs elicited by dichoptic stimulation in children with normal and compromised stereopsis (due to amblyopia). **Methods:** Two groups of six children (age- and sex-matched: 3M/3F aged 7–12 yo), one with compromised stereopsis and one with normal stereopsis, were included. OFRs were recorded using a custom high-resolution video eye-tracking system. The relative differences between eye displacement induced by correlated stimuli (up-correlated–down-correlated) and anticorrelated (up-anticorrelated–down-anticorrelated) were compared. **Results:** We found significant differences between OFRs induced by two dichoptic conditions (correlated and anticorrelated stimuli) in most children with normal stereopsis, whereas no differences were observed in children with compromised stereopsis, indicating a lack of disparity detectors. **Conclusions:** OFRs might thus be exploited as a diagnostic tool for the objective identification of stereo deficiencies in children. This might lead to improved early diagnosis and treatment outcomes for conditions like amblyopia and strabismus.

## 1. Introduction

Having two eyes as opposed to one eye has at least three advantages: (1) it provides redundancy in the case of injury to one eye; (2) it expands the size of the visual field that can be taken in with one fixation; and (3) it enables animals with overlapping monocular visual fields to extract depth information by comparing the images from two eyes [1]. Binocular single vision (BSV) refers to the ability to perceive a single visual image by fusing the images from the two eyes. This occurs within a limited depth range, called Panum’s area, around the point at which the two lines of sight intersect. Depth perception, also known as stereopsis, is then mediated by binocular disparities: An object whose image falls at different retinal loci on the two retinas will be seen as being outside of the plane of fixation [2]. Stereopsis within the range of small disparities that fall within Panum’s area is usually referred to as “fine”. Stereopsis is, however, also supported by disparities so large that stimuli appear diplopic (coarse stereopsis) [3]. 

In primates and other frontal-eyed mammals, monocular signals are kept separate in the lateral geniculate nucleus [4] and in the input layer of the primary visual area (V1) [5]. These inputs are combined at the next V1 synapse [6,7,8,9]. The available evidence suggests that cortical binocular cells mediate BSV and the associated sensation of depth [10,11]. The development of such cells requires normal visual experience [12,13]. Subjects with strabismus (the misalignment of the two eyes) invariably have no or very poor disparity-based depth perception [14,15,16]. The idea that cortical binocular neurons are crucial for stereopsis is then supported by the observation that the sensitivity of cortical cells to binocular disparity is much reduced in cats [17,18,19,20] and monkeys [21,22,23,24,25] reared with strabismus.

It is well established that proper development of stereopsis requires binocular alignment and similar image clarity in both eyes in childhood [26,27,28]. There is still controversy about the exact critical period: It is generally accepted that it includes the period between 3 months and 2 years of age [29], although it might extend up to 5 years of age, by which time coarse stereopsis has fully matured [30]. In children with normal stereopsis, fine stereopsis continues to mature into the school-age years (6–9 years). In addition to strabismus, which in Western societies has an incidence of 2–4% [31,32], amblyopia is the other major cause of poor stereopsis. It is a deficit in visual acuity (usually monocular, but occasionally binocular) that is resistant to refractive correction. It is caused by strabismus, a large refractive difference between the eyes (anisometropia), the presence of deprivation factors, or a combination of these factors [16]. In Western societies, it has an incidence of 1–2% [32,33] and it often disrupts fine stereopsis [34,35], particularly when strabismus is also present [36,37]. Its critical period extends from birth to 7–8 years of age [38].

Stereo deficiencies, defined as pathological conditions that result in no or very limited stereoscopic perception, have been estimated to affect as much as 7% of the population [39]. Although late-onset cases do occur, especially following trauma, stereo deficiency is usually a developmental central nervous system disorder due to the brain’s inability to merge inputs from the two eyes. Over time, this often leads to favoring one eye or alternating between eyes [40]. Medical interventions (such as eye muscle surgery, orthoptics, eye patching, and exposure to enriched visual experience) can lead to an improvement in stereopsis. Because the efficacy of therapy decreases with age, early diagnosis and intervention are desirable [24,30,41,42,43].

Several clinical tests to assess binocular depth perception are already available, with varying levels of specificity and sensitivity [44,45]. Unfortunately, they all require patient cooperation and are thus better suited for school-age children and adults [37,46,47]. It would thus be desirable to develop a test capable of directly detecting the improper development of cortical binocular cells without having to rely on what a subject perceives. 

Ocular-following responses (OFRs) are reflexive, short-latency eye movements induced by the sudden motion of a large textured pattern in the visual field [48]. While their functional role has not been firmly established, they might represent the initial component of the translational vestibulo-ocular reflex and thus contribute to the stabilization of the gaze [49,50,51,52,53]. Lesion studies in monkeys revealed that they are controlled by the cortical dorsal visual pathway, from V1 to the middle superior temporal (MST) area [54]. Like many other types of eye movements, they are mostly conjugate, in that both eyes move at the same speed and in the same direction whether the visual stimulus is seen monocularly or binocularly. However, unlike with other conjugate eye movements, binocular stimulation induces much larger OFRs than monocular stimulation [55]. Because they are driven by cortical neurons, which as noted above are almost invariably sensitive to binocular disparity, they might be sensitive to interocular correlations. Using dichoptically presented drifting random line stimuli, Quaia and colleagues [55,56] showed that in stereo-competent subjects, OFRs are larger when the two eyes see the same pattern than when they see two different (uncorrelated) patterns. OFRs are even smaller when the pattern seen by one eye is the contrast-reversed version of the one seen by the other eye (anticorrelated). The sensitivity of cortical neurons to interocular correlations is thus reflected in the OFRs. Neurophysiological studies have shown that in amblyopic and strabismic monkeys (and presumably in stereo-blind subjects in general), cortical neurons have a very low sensitivity to binocular correlations. It thus follows that the sensitivity to interocular correlations seen in the OFRs of stereo-competent subjects should be much attenuated in stereo-blind subjects. A study comparing OFRs induced by correlated (i.e., identical in the two eyes), uncorrelated, and anticorrelated stimuli in adult healthy and strabismic subjects has shown just that. OFRs in strabismic subjects are insensitive to interocular correlations [56]. Thus, these eye movements have the potential to serve as a tool for detecting stereo anomalies alternative to those that rely on perceptual reports. Importantly, their reflexive nature minimizes the need for subject cooperation, making them appealing in non-verbal and child populations.

While promising, there is a caveat: because of their small amplitudes, OFRs are usually recorded using scleral search coils and are thus confined to few research settings and are not suitable for use in children. In a recent study [57], we successfully measured OFRs in children using a high-resolution video eye tracker during 3 min recording sessions, demonstrating that it is possible to record OFRs accurately and non-invasively in children in a clinical setting. 

This study aims to compare the OFRs induced by correlated and anticorrelated stimuli in a small group of healthy and amblyopic children, so as to evaluate the potential of this method to detect stereo deficiencies.

## 2. Materials and Methods

### 2.1. Study Population

This study is part of a larger project on the potential clinical applications of reflexive eye movements. OFRs were recorded at the Ophthalmology Department of the Institute for Maternal and Child Health-IRCCS Burlo Garofolo (Trieste, Italy). Two groups of six children (age- and sex-matched: 3M/3F aged 7–12 yo), one with compromised stereopsis and one with normal stereopsis were selected for this study. All children underwent a complete ophthalmological and orthoptic examination, including measurement of logarithm of the minimum angle of resolution visual acuity (logMAR), cycloplegic refraction, detection of horizontal and/or vertical manifest strabismus angle by means of prisms, and stereoacuity measurement using the Titmus stereo test. This test probes local stereopsis by testing the ability to match similar image features in the two eyes and is easier to use in children than tests for global stereopsis (which use random dot stereograms, like TNO). Normal disparity levels in 3–4-year-old children are 100″ when evaluated with the Titmus test [58]. The level associated with the coarsest stereopsis (800″) can be passed by strabismic adults and children and also by children with monofixation syndrome. Patients with poor vision are also able to pass the test if the largest disparity is used, but not with small disparities (40–140″) [59]. 

The inclusion criteria for healthy subjects were a better than 0.0 logMAR without correction, a hyperopic cycloplegic refraction between 0.50 and 2.00 diopters (D), no astigmatism or anisometropia, and stereo acuity < 100″. Amblyopic subjects were selected among patients followed by the orthoptics and amblyopia rehabilitation center of our hospital. Inclusion criteria were one eye with a 0.0 logMAR and the other of 0.2–0.4 logMAR, horizontal strabismus angle < 12 prismatic diopters (PDs) and vertical < 5 PDs, and stereo acuity > 400″. Exclusion criteria were organic visual loss and any other cause of visual impairment in addition to refractive and strabismic amblyopia. Complete demographic and clinical information of the healthy and amblyopia cohort is reported in Table 1. All patients, except P3 who presented a refractive anisometropic amblyopia, had a small angle of strabismus (exotropia in P1; esotropia in P2, P5, and P6; and right hypertropia in P4) subsequent to strabismus surgery performed a minimum of one year before the experiment. All the subjects who underwent strabismus surgery had infantile essential esotropia with a strabismus angle higher than 30 prismatic diopters and underwent surgery between the ages of 3 and 4.

### 2.2. OFR Recording Apparatus

We recorded OFRs using a custom high-resolution video eye-tracking system that we previously developed for this purpose. Images were acquired using a high-resolution camera (Grasshopper 3 GS3-U3-51S5M-C, Teledyne FLIR LLC, Wilsonville, OR, USA) with a resolution of 2448 × 2048 pixels, equipped with a C-Mount 50 mm f/2.8 lens. 

**Figure 1:**
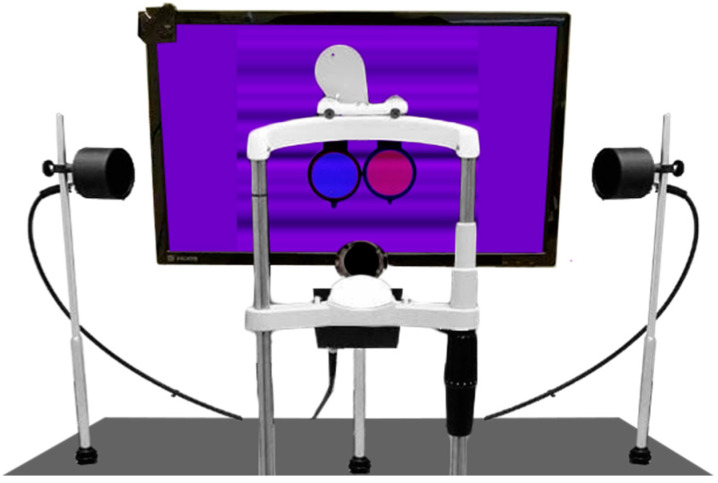
The custom designed high-resolution eye-tracking system used for measuring OFRs. Color filters are placed in front of the eyes, so that each eye sees only the red or blue video channel (dichoptic stimulation). The green monitor channel is not used, so that all images would appear purple if seen binocularly.

Because the pupil is more easily isolated from the iris in the near-infrared than in the visible spectral range, a Hoya R72 IR filter was placed in front of the camera lens, thus suppressing wavelengths shorter than 720 nm. To ensure optimal lighting conditions, we used three custom-built infrared (IR) LED illuminators: one on each side of the subject and one in front and below the subject, as illustrated in Figure 1.

Subjects were seated in a dimly lit room with their head gently stabilized using padded chin and forehead supports. They faced a monitor (VG248QE, ASUSTeK Computer Inc., Taipei, China) placed at a distance of 50 cm from the corneal vertex. The monitor was set at a resolution of 1920 × 1080 pixels and at a vertical refresh rate of 144 Hz. The chair height was adjusted to align the subjects’ eyes with the screen’s center. To independently control the luminance pattern seen by each eye (dichoptic presentation), we created colored images in which the blue and the red channels contained the patterns to be seen by each eye. A different colored lens was then placed in front of each eye: one let through only wavelengths shorter than 550 nm, appearing blue, whereas the other let through only wavelengths longer than 550 nm, appearing red. Blue and red were selected because they are far apart in the color spectrum and thus are more effectively separated by the color filters. Green would be seen through both filters, but in all our stimuli, the green channel was always set to zero luminance.

To track movements of the head, we placed a black marker close to the bridge of the nose of the subject. The camera was then oriented so that each image contained the head marker and the eye that saw the red channel (IR light was blocked by the blue lens in front of the other eye).

### 2.3. Behavioral Paradigm

**Figure 2:**
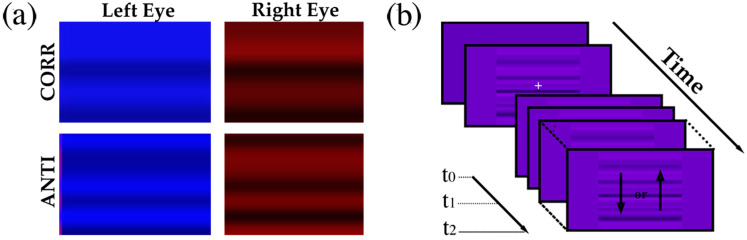
(**a**) Binocularly correlated (upper) and anticorrelated stimuli (lower) were presented by separately controlling the red and blue channels and by placing a red filter in front of the red eye and a blue filter in front of the left eye; (**b**) stimuli drifted (at a speed of 50°/s) upward or downward for a duration of 200 ms.

Each trial started with a blank screen at mid-luminance (7.0 cd/m^2^ through each lens). Without colored lenses, it would appear purple (at 14 cd/m^2^) because only the red and blue channels were used (see Figure 2b). A fixation cross was then displayed in the center of the screen, seen only through the red channel. The only instruction given to the subject was to stay as still as possible and to keep staring at the fixation cross. After a short variable interval (1–1.5 s), the fixation cross disappeared and a dichoptic stimulus was presented and started to drift immediately. Stimuli were presented within a static square aperture (28 deg side) centered on the screen. They were generated by randomly assigning high or low luminance values (symmetric around the mean luminance, which was set to 7.0 cd/m^2^ like the background) to consecutive pairs of pixel rows (0.06 deg). The resulting 1D random line stimulus was then low-pass filtered with a digital filter having zero gain above 0.75 cycles per degree (cpd) and a gain of one below 0.375 cpd (following a raised-cosine function transition). The root mean square contrast of the stimulus was set at 30% to prevent saturation. To simulate motion, the 1D pattern drifted either upward or downward at a speed of approximately 50°/s, appearing to move behind a static aperture. The responses to two stimulus conditions were investigated. In the first condition (correlated), the images seen by the two eyes were identical and thus had zero disparity and an interocular correlation of 1.0 (as shown in Figure 2a, upper image). In the second condition (anticorrelated), the image shown to one eye was created by contrast-reversing the one shown to the other, resulting in a binocular stimulus with zero disparity and an interocular correlation of −1.0 (as shown in Figure 2a, lower image). Correlated and anticorrelated stimuli thus shared identical spatial and temporal frequency contents and contrasts and differed exclusively in interocular correlation. In each of the two conditions, the images could drift either up or down, but always in the same direction in the two eyes. During each trial, we acquired three frames at specific time points: t_0_ = 0 ms (fixation cross offset/stimulus onset), t_1_ = 80 ms (the typical ocular-following latency in humans for stimuli of comparable size and contrast as determined in prior studies [60,61]), and t_2_ = 160 ms (the end of the open-loop phase of the movement). 

Within an experimental session, a total of 120 trials, organized into 30 blocks of stimuli for each condition, were presented to the subjects.

### 2.4. Data Analysis

Camera images (three for each trial) were stored on disk, and extraction of OFR measurements was carried out off-line. The first step of the analysis consisted in extracting the subpixel displacement of the center of the imaged pupil and of the head marker across two time windows: the fixation epoch (comparing frames recorded at times t_1_ vs. t_0_) and the movement epoch (comparing frames recorded at times t_2_ vs. t_1_). Trials in which the subject blinked or made such a large head movement that either the pupil or the head marker exited the frame were discarded at this stage, accounting for as many as 30% of the trials in some subjects.

To estimate eye displacement (eye-in-head) during the fixation and movement epochs, we then subtracted the head marker displacement (head-in-space) from the pupil displacement (eye-in-space) in the respective epoch. An outlier detection procedure was applied to automatically exclude from the analysis trials in which fixation was poor (for example, because of the presence of saccades, microsaccades, or large head movements). The goal was to discard trials in which either the head or the eyes moved too much during the fixation period, or the head moved too much during the movement period. We defined “too much” as a deviation from the median value by more than three times the robust estimate of the standard deviation, repeated iteratively until no trials were discarded any more. On average, this procedure resulted in excluding 20% of the trials (ranging from 5% to 45% across subjects). 

With the remaining trials, we then separately computed the OFRs as the difference of the vertical displacements in the movement window induced by upward- and downward-drifting stimuli for correlated and anticorrelated conditions. To test for significant differences between upward and downward movements, we used the unpaired *t*-test and Mann–Whitney U test, with a 0.05 significance level. The relative differences between eye displacement induced by correlated stimuli (up-correlated–down-correlated) and anticorrelated (up-anticorrelated–down-anticorrelated) were compared using a bootstrap method. All statistical analyses were performed in Python using the statsmodels and scipy packages. 

## 3. Results

The mean and SD values of the vertical displacements in the open-loop period (t_2_–t_1_) induced by upward- and downward-drifting stimuli for correlated and anticorrelated conditions are reported in Table 2 and Table 3, respectively. In both groups, we found that upward- and downward-moving stimuli induced significantly different eye displacements for both binocularly correlated (Table 2) and anticorrelated stimuli (Table 3).

**Figure 3:**
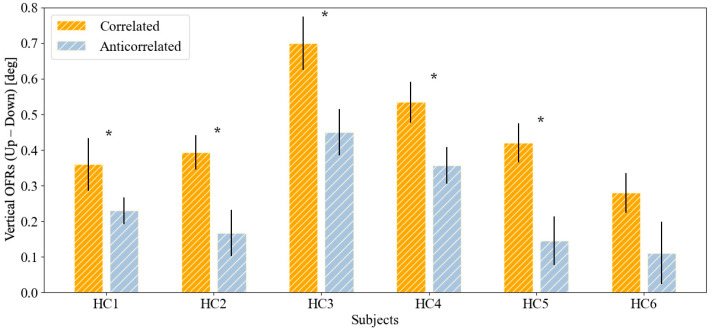
Bar plots with standard error whiskers of the mean OFRs induced by two conditions in subjects with normal stereopsis (HC1–HC6). The asterisks denote a statistically significant difference between OFRs induced by correlated and anticorrelated stimuli.

**Figure 4:**
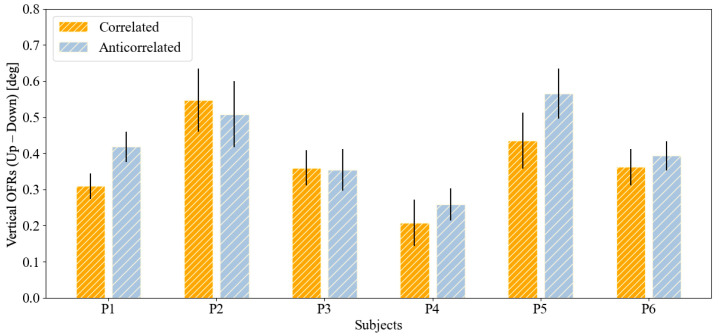
Bar plots with standard error whiskers of the mean OFRs induced by two conditions in subjects with compromised stereopsis (P1–P6). No statistically significant differences were observed.

The mean OFRs induced by the two stimuli in subjects with normal stereopsis (HC1–HC6) and in subjects with compromised stereopsis (P1–P6) are reported in Figure 3 and Figure 4, respectively. In five out of six subjects with normal stereopsis, the responses to correlated stimuli were significantly larger than those to anticorrelated stimuli (Figure 3). In contrast, none of the subjects with compromised stereopsis showed a statistically significant difference between the magnitude of responses to correlated and anticorrelated stimuli (Figure 4). In these subjects, the OFRs were thus not sensitive to interocular correlation. A summary of the numerical values plotted in these figures can be found in Table 4. 

**Figure 5:**
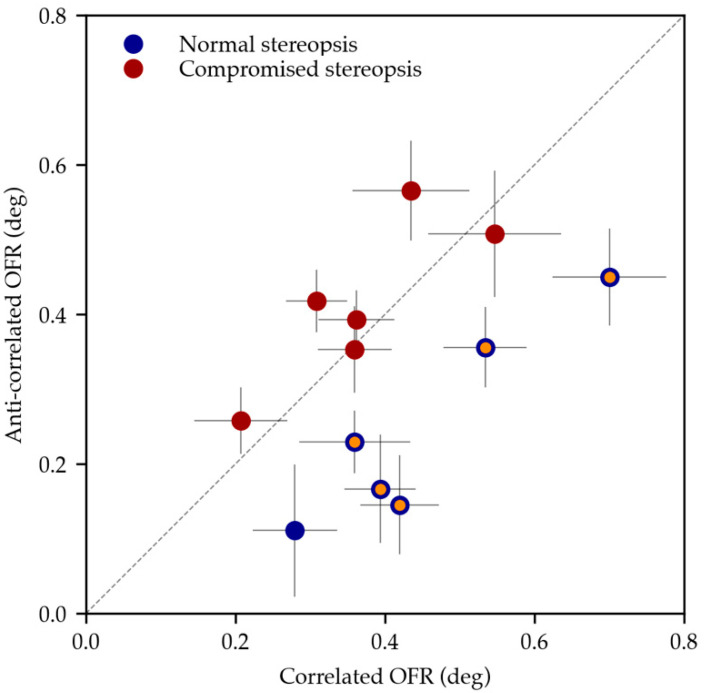
Comparison between OFRs induced by correlated (x-axis) and anticorrelated (y-axis) stimuli in subjects with normal stereopsis (blue) and compromised stereopsis (red). Statistically significant differences between OFRs induced by the two conditions are highlighted in orange.

In Figure 5, we summarize our results by plotting the OFRs induced by correlated and anticorrelated stimuli for all subjects. There is considerable variability in the absolute magnitude of the responses to the correlated stimuli in both groups. Unlike stereo-deficient subjects, healthy subjects tend to have smaller responses to anticorrelated stimuli compared to the correlated stimuli, and thus their data points mostly lie below the identity line. 

## 4. Discussion

In this study, we investigated whether it is feasible to use OFRs induced by correlated and anticorrelated stimuli to detect stereo deficiency in children. The most common causes of amblyopia are strabismus, a refractive difference between the eyes (anisometropia), or both, in early childhood. We chose this pathology for our positive cohort because stereopsis is often disrupted in amblyopia, particularly when associated with strabismus. 

In both children exhibiting normal stereopsis and those with compromised stereopsis, we observed statistical differences in eye displacements induced by stimuli moving upward and downward during the open-loop period (Table 2 and Table 3). This phenomenon was evident for both binocularly correlated and anticorrelated stimuli, indicating the presence of detectable OFRs in both groups, as reported in our previous studies [57,62]. The magnitude of the responses fell within the range we previously reported using different drifting stimuli [57]. This reaffirms the presence of OFRs in school-age children irrespective of their stereoscopic vision status, serving as a foundational basis for utilizing OFRs as a tool to investigate interocular sensitivity.

Our experiment reveals statistically significant differences in five out of six healthy children, while in all children with compromised stereopsis, no significant differences were observed. Thus, in our small cohort, sensitivity was high and specificity was acceptable, indicating that studying a larger population is warranted. 

The lack of false negatives was not particularly surprising. The only way for the brain to differentiate between our correlated and anticorrelated stimuli is if cells sensitive to binocular disparity are present. The p-value chosen for the statistical test thus probably represents an upper limit on false negatives. False positives are instead a more important problem, and they could conceivably arise in many ways. First of all, we noted that there is a large variability across subjects in the magnitude of the OFRs [57], but the noise level does not covary with it. Hence, the likelihood of detecting a certain percentage difference in response to correlated vs. anticorrelated stimuli decreases with the size of the OFRs. For example, subject HC6, our false positive, had the smallest OFRs in our normal cohort. From our clinical evaluation, this appears to be a true false positive, i.e., a healthy subject that is flagged by the test as anomalous. Recording the OFRs in a large group of subjects will be necessary to estimate the rate at which this can be expected to occur. If this rate were too high, the development of an even higher resolution video eye-tracking system (e.g., based on the iris instead of pupil tracking) might be necessary. But small OFRs are not the only potential source of false positives. Consider, for example, the case in which a subject is effectively blind in one eye. Then, both correlated and anticorrelated stimuli would be seen monocularly, and thus as identical, and would necessarily generate identical OFRs (i.e., a positive result). Alternatively, suppose that a subject had large vertical strabismus. Then, a large disparity would be added to the correlated stimuli, possibly bringing them outside the range covered by disparity detectors, thus yielding a positive result not necessarily based on the lack of disparity detectors [63,64,65]. Obviously, seeing through only one eye and having large vertical strabismus would still be pathological conditions, and thus a positive result, even when not the result of a direct test of binocular disparity detectors, would still warrant additional attention from the clinician and should not be considered a false positive.

To minimize such trivial false positives, in our study, we focused on cases of amblyopia associated with mild anisometropia and/or small-angle strabismus (following eye muscle surgery) that had already been treated with no success. Furthermore, because all but one of our amblyopic subjects had only small-angle horizontal strabismus, we used horizontal line stimuli, limiting interocular correlations with the vertical direction. Because horizontal strabismus does not introduce any vertical disparities with such a stimulus, interocular correlations are preserved. For this same reason, this stimulus is also robust to convergence insufficiency (fairly common given the short distance to the monitor [63]) and unmasked phorias, which tend to be mostly horizontal [64,65].

Our subjects were school-age, verbal, and cooperative, allowing for simpler tests to be used to carry out this assessment. However, the existing tests, which are all based on testing stereopsis (perception of depth based on horizontal binocular disparities), have important limitations. First, some of them can be solved using monocular cues. Those that rely on the detection of disparities of the outline of simple stimuli (probing local stereopsis), such as the Titmus test, can yield false negatives, especially when the viewing angle is not strictly controlled (as it is often difficult to accomplish in young children). As a consequence of its 3D construction, the Frisby test also exhibits the problem of motion parallax. Other methods, such as the Lang and the TNO tests, are instead based on solving a complex binocular correspondence problem between fields of random dots (probing so-called global stereopsis). This is a much stricter test of stereopsis, insensitive to monocular cues, and it can only be solved through binocular-selective cells. However, it is still an indirect test of the proper development of binocular disparity cells, because it requires an understanding of the task by the subject and a verbal report, which cannot be taken for granted in young children [66]. 

Recording OFRs is undoubtedly much more cumbersome than any of the current stereopsis tests and currently beyond routine clinical practice. However, it has many theoretical advantages over other stereopsis tests. First, the OFRs are directly generated by neurons that are sensitive to (absolute) binocular disparity and do not rely on the extraction of the relative horizontal disparities that underlie stereopsis. A case in point is that our stimuli actually have zero horizontal disparity; they do not generate any depth perception. Second, with our horizontal 1D line stimuli, the neurons that drive OFRs are tuned to vertical binocular disparities, which do not support stereopsis but only (vertical) disparity vergence. Finally, taking advantage of the sensitivity to interocular correlation of a conjugate eye movement has the added advantage that a stereo anomaly can be detected by recording the movement of a single eye.

## 5. Conclusions

We recorded OFRs in children, focusing on their responses to dichoptic stimulation and their potential for assessing stereo deficiencies associated with conditions like amblyopia. The results reveal that correlated stimuli induced substantially larger OFRs than anticorrelated stimuli in most healthy, but not in stereo-deficient children. These findings suggest that OFRs could potentially serve as a diagnostic tool for identifying stereo deficiencies. Future research with larger sample sizes will be required to establish the value of this approach for everyday clinical applications. 

## Figures and Tables

**Table 1:** Clinical and demographic information of children with normal (HC1–HC6) and compromised stereopsis (P1–P6). (logMAR: logarithm of the minimum angle of resolution; LE: left eye; RE: right eye; PD: prism diopter; XT: exotropia; ET: esotropia; and HYTR: hypertropia).

Subj	Age	Sex	Cycloplegic Refraction		LogMAR		Strabismus Angle
			Right Eye (RE)	Left Eye (LE)	RE	LE	(PD)
HC1	7	M	-	-	≤0.0	≤0.0	-
HC2	8	F	-	-	≤0.0	≤0.0	-
HC3	9	F	-	-	≤0.0	≤0.0	-
HC4	9	M	-	-	≤0.0	≤0.0	-
HC5	11	M	-	-	≤0.0	≤0.0	-
HC6	12	F	-	-	≤0.0	≤0.0	-
P1	7	M	+1.25 + 0.50/80	+1.50	0.2	0.0	6 XT
P2	8	F	+2.00	+2.25	0.0	0.4	10 ET
P3	9	F	+2.25 + 1.00/80	+3.50 + 2.50/80	0.0	0.4	-
P4	9	M	+0.50	+0.50	0.2	0.0	RE HYTR 4
P5	11	M	+3.00 + 0.50/95	+3.25 + 1.50/105	0.0	0.3	8 ET
P6	12	F	+2.25 + 0.50/95	+2.25 + 0.50/75	0.0	0.2	10 ET

**Table 2:** Eye displacement during the movement window (80–160 ms from motion onset) induced by binocularly correlated stimuli (HC#: healthy control; P#: patient; Δyc: vertical eye displacement (deg); Negative values indicate downward movement; N: number of correct trials; *p*: unpaired *t*-test; p_np: Mann–Whitney U test).

Subj	Δyc ± SD (N) UP	Δyc ± SD (N) DW	*p*	p_np	OFRc ± SD
HC1	0.227 ± 0.234 (12)	−0.133 ± 0.096 (13)	<0.001	<0.001	0.360 ± 0.253
HC2	0.220 ± 0.088 (6)	−0.174 ± 0.105 (13)	<0.001	<0.001	0.394 ± 0.137
HC3	0.424 ± 0.157 (10)	−0.277 ± 0.183 (11)	<0.001	<0.001	0.700 ± 0.241
HC4	0.293 ± 0.101 (13)	−0.242 ± 0.186 (15)	<0.001	<0.001	0.534 ± 0.212
HC5	0.216 ± 0.131 (12)	−0.204 ± 0.132 (11)	<0.001	<0.001	0.420 ± 0.186
HC6	0.157 ± 0.125 (16)	−0.123 ± 0.152 (11)	<0.001	<0.001	0.280 ± 0.197
P1	0.192 ± 0.119 (18)	−0.116 ± 0.104 (18)	<0.001	<0.001	0.309 ± 0.158
P2	0.124 ± 0.177 (14)	−0.423 ± 0.276 (14)	<0.001	<0.001	0.547 ± 0.328
P3	0.187 ± 0.104 (12)	−0.173 ± 0.117 (9)	<0.001	<0.001	0.360 ± 0.157
P4	0.090 ± 0.184 (12)	−0.117 ± 0.141 (16)	0.003	0.003	0.207 ± 0.232
P5	0.278 ± 0.136 (11)	−0.157 ± 0.216 (10)	<0.001	0.001	0.435 ± 0.255
P6	0.126 ± 0.101 (14)	−0.236 ± 0.176 (17)	<0.001	<0.001	0.362 ± 0.202

**Table 3:** Eye displacement during the movement window (80–160 ms from motion onset) induced by binocularly anticorrelated stimuli (HC#: healthy control; P#: patient; Δyac: vertical eye displacement (deg); Negative values indicate downward movement; N: number of correct trials; *p*: unpaired *t*-test; p_np: Mann–Whitney U test).

Subj	Δyac ± SD (N) UP	Δyac ± SD (N) DW	*p*	p_np	OFRac ± SD
HC1	0.102 ± 0.111 (9)	−0.127 ± 0.041 (12)	<0.001	<0.001	0.230 ± 0.118
HC2	0.168 ± 0.175 (11)	0.001 ± 0.149 (12)	0.028	0.006	0.167 ± 0.230
HC3	0.158 ± 0.120 (08)	−0.291 ± 0.171 (12)	<0.001	<0.001	0.450 ± 0.209
HC4	0.232 ± 0.154 (10)	−0.124 ± 0.049 (6)	<0.001	<0.001	0.356 ± 0.161
HC5	0.100 ± 0.165 (15)	−0.045 ± 0.188 (14)	0.042	0.017	0.145 ± 0.250
HC6	0.158 ± 0.086 (16)	0.047 ± 0.289 (12)	0.172	0.024	0.111 ± 0.301
P1	0.163 ± 0.115 (17)	−0.255 ± 0.141 (22)	<0.001	<0.001	0.418 ± 0.182
P2	0.155 ± 0.256 (12)	−0.353 ± 0.172 (13)	<0.001	<0.001	0.508 ± 0.308
P3	0.143 ± 0.132 (9)	−0.211 ± 0.127 (10)	<0.001	0.001	0.354 ± 0.183
P4	0.131 ± 0.132 (13)	−0.127 ± 0.091 (15)	<0.001	<0.001	0.258 ± 0.160
P5	0.288 ± 0.138 (6)	−0.278 ± 0.094 (7)	<0.001	0.001	0.566 ± 0.167
P6	0.187 ± 0.104 (17)	−0.206 ± 0.117 (14)	<0.001	<0.001	0.393 ± 0.157

**Table 4:** The mean (deg), standard deviation, and standard error (SEM) of OFRs elicited by correlated stimuli (OFRc) and anticorrelated stimuli (OFRac) were computed for individuals with intact stereopsis (subjects HC1–HC6) and those with impaired stereopsis (subjects P1–P6). Asterisks denote statistically significant differences between the OFRs induced by these two conditions.

Subj	OFRc ± SD	OFRc SEM	OFRac ± SD	OFRac SEM	*p*
HC1	0.360 ± 0.253	0.073	0.230 ± 0.118	0.038	0.048 *
HC2	0.394 ± 0.137	0.045	0.167 ± 0.230	0.064	0.010 *
HC3	0.700 ± 0.241	0.073	0.450 ± 0.209	0.065	0.016 *
HC4	0.534 ± 0.212	0.057	0.356 ± 0.161	0.054	0.014 *
HC5	0.420 ± 0.186	0.056	0.145 ± 0.250	0.065	<0.001 *
HC6	0.280 ± 0.197	0.054	0.111 ± 0.301	0.084	0.088
P1	0.309 ± 0.158	0.038	0.418 ± 0.182	0.040	0.092
P2	0.547 ± 0.328	0.088	0.508 ± 0.308	0.089	0.738
P3	0.360 ± 0.157	0.049	0.354 ± 0.183	0.059	0.936
P4	0.207 ± 0.232	0.065	0.258 ± 0.160	0.043	0.554
P5	0.435 ± 0.255	0.079	0.566 ± 0.167	0.069	0.184
P6	0.362 ± 0.202	0.049	0.393 ± 0.157	0.042	0.632

## Data Availability

The raw data consist of facial images of the children, which, in compliance with privacy restrictions, cannot be publicly disclosed.
